# Modeling lifetime abuse and cardiovascular disease risk among women

**DOI:** 10.1186/s12872-019-1196-y

**Published:** 2019-10-16

**Authors:** Kelly A. Scott-Storey, Marilyn Hodgins, Judith Wuest

**Affiliations:** Faculty of Nursing, University of New Brunswick, P.O. Box 4400, Fredericton, New Brunswick E3B 5A3 Canada

**Keywords:** Women, Cardiovascular disease risk, Depression, Lifetime violence, Structural equation modeling

## Abstract

**Background:**

Cardiovascular disease (CVD) is one of the most significant health challenges facing women today. Abuse is a serious gendered issue also affecting the health of women. Despite beginning evidence that abuse may increase the risk of CVD among women, causal pathways linking abuse to CVD have received little attention. Our purpose was to test Scott-Storey’s conceptual model showing direct and indirect pathways through which lifetime abuse severity may affect women’s CVD risk.

**Methods:**

Using data collected from a community sample of 227 Canadian women who had left an abusive partner, we conducted structural equation modeling with latent growth curve analysis using a phantom variable approach to test the direct effects of severity of lifetime abuse on CVD risk (indicated by measures of systolic and diastolic blood pressure) as well as its indirect effects through CVD risk behaviors and through women’s initial level of depressive symptoms and the observed rate of change in their depressive symptoms over time.

**Results:**

Women in this sample had above average CVD risk factors (i.e., smoking, overweight/obesity, depressive symptoms, high blood pressure) in comparison to women in the general population. Further, CVD risk behaviors increased with severity of lifetime abuse and remained present long after leaving the abusive relationship. Results of the tested model provide preliminary evidence supporting many of the hypothesized pathways by which severity of lifetime abuse can increase CVD risk among women; the model fit the data reasonably well explaining 41% of the variance in CVD risk.

**Conclusions:**

Findings support the growing recognition of the long-term effects of lifetime abuse on cardiovascular health, suggest important implications for clinicians working with women, and provide a novel approach for studying the concept of cumulative lifetime abuse through the use of a phantom variable.

## Background

Abuse against women is globally recognized as a gendered issue and a major public health concern disproportionally affecting one-quarter to one-half of all women within their lifetime [1–4]. Abuse is a major determinant of women’s health having been found to be associated with long-lasting deleterious mental and physical health effects that persist even after the abuse has ended [4–10]. Recently, cardiovascular disease (CVD) has been identified as a long-term consequence of abuse among women [11–14]. This link is important because CVD is the greatest cause of premature mortality among women, affecting 1 in 3 in North America [15, 16]. For those living with CVD, the chronicity of the disease translates into substantial disability [16, 17]. In developed countries, CVD is currently one of the most expensive health problems and its economic burden is expected to worsen [16, 18].

To date, much of the research examining the link between abuse and CVD has relied on correlational and regression analyses, which fails to explore the causal pathways between abuse and CVD. Understanding *how* abuse affects the risk for CVD is imperative for informing prevention and intervention efforts among vulnerable women, yet few studies have explicitly tested these causal pathways with mediation models [10] To address this gap, Scott-Storey [19] developed a conceptual model depicting direct and indirect pathways by which severity of lifetime abuse may affect women’s CVD risk. The purpose of this study was to test Scott-Storey’s conceptual model.

### Conceptual model

Abuse refers to the “intentional use of physical force or power, threatened or actual, against another person that either results in or has a high likelihood of resulting in injury, death, psychological harm, maldevelopment or deprivation” [20]. Abuse experiences for women include a range of abuse types (e.g., physical, sexual, psychological) that co-occur and re-occur across the lifespan (e.g., childhood abuse, intimate partner violence, adult sexual assault) [21]. The severity of cumulative experiences of abuse has been associated with incrementally worse health outcomes, yet the majority of abuse-related research has focused on specific types of abuse or abuse at distinct points in the lifespan, leaving important gaps in our understanding of the cumulative impact of severity of *lifetime abuse* on health. For a more in-depth review of the conceptualization, operationalization and methodological approaches to understanding cumulative abuse in relation to women’s health refer to Scott-Storey [21].

**Fig. 1:**
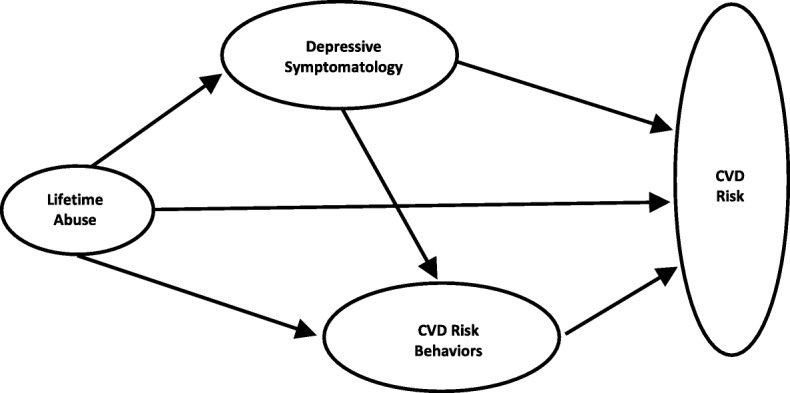
Conceptual model of the pathways through which lifetime abuse increases CVD risk. From K. Scott-Storey, Abuse as a gendered risk factor for cardiovascular disease: A conceptual model, J Cardiovasc Nurs. 2013; 28(6)p. E2. doi: 10.1097/JCN.0b013e318279e372 Copyright by Wolters Kluwer Health, Inc. Reprinted with permission

Little attention has been given to the development of conceptual models to explain how lifetime abuse influences CVD risk among women. Drawing on stress theory and empirical evidence, Scott-Storey’s conceptual model (Fig. 1) delineates three pathways through which lifetime abuse may increase CVD risk among women. First, *lifetime abuse* is positioned as a chronic stressor, potentially causing significant neuroendocrine, metabolic, haemostatic, and immunologic changes within the body. These physiological changes are believed to create a state of vulnerability, leading to the etiology of CVD [22]. Specifically, chronic stress from abuse can lead to elevated blood pressure, a clinically important early indicator of CVD risk [19]. Second, common coping strategies for dealing with stress from abuse, such as smoking and overeating, are known *CVD risk behaviors* [16, 23, 24]. These risk behaviors often persist long after the abuse has ended, and may be an indirect pathway through which abuse contributes to CVD risk. A third pathway is through *depressive symptoms*. Women with histories of abuse have higher incidences of depressive disorders and depressive symptoms [25–28]. Depression may be partially the consequence of physiological abuse-related changes in the brain that ultimately impact behavior and mood [29, 30]. The severity of depressive symptoms has been linked to worse hypothalamic-pituitary-adrenal (HPA) axis dysregulation [31–33] and more significant physiological changes in the brain [34]. In turn, depressive disorders and depressive symptoms have been associated with CVD [35, 36]. Depressive symptoms are also associated with engagement in CVD risk behaviors such as smoking and overeating [10, 23, 37]. Therefore, depressive symptoms are expected to contribute to CVD risk, as well as to engagement in CVD risk behaviors of smoking and overeating, which in turn affect CVD risk.

Despite the established association between depression and CVD, little is known about the depressive symptoms experienced by women once they leave an abusive partner or how changes in depressive symptoms over time may affect health and/or risk behaviors. Because depression results in chronically elevated levels of cortisol, catecholamines, and inflammatory markers, all of which promote the development and progression of CVD [10, 31, 37, 38], we hypothesized that it is the change in women’s depressive symptoms after leaving an abusive partner, rather than the level of depressive symptoms at the time of leaving, that predominantly influences CVD risk.

Depression may indirectly affect CVD due to its effect on CVD risk behaviors as smoking and overeating offer a way to cope with the negative emotions evoked by stress and depression [14, 39–41]. These coping behaviors often persist, and may even increase, because stress does not abate after leaving [6, 42]. These risk behaviors may be compounded if a woman is concurrently suffering from depressive symptoms [43–45]. Thus, we hypothesized that severity of depressive symptoms in the years after leaving an abusive partner will affect behaviors of smoking and overeating long after separating. In addition, change in depressive symptoms overtime will influence these behaviors. For example, if depressive symptoms worsen, over-eating is likely to occur resulting in weight gain.

In summary, we aimed to test a conceptual model hypothesizing that severity of lifetime abuse increases CVD risk, as indicated by blood pressure, among women directly as well as indirectly through: a) CVD risk behaviors of smoking and over-eating, and b) both the initial level and the rate of change in depressive symptoms over time.

## Methods

We analysed existing longitudinal quantitative data from the Women’s Health Effects Study, a prospective study examining patterns of women’s mental and physical health in the early years after leaving an abusive male partner [8]. The community-based sample consisted of 309 English-speaking women 19 years of age and older from three Canadian provinces (New Brunswick, Ontario and British Columbia) who had left an abusive partner from 3 months to 3 years prior to enrollment in the study. Data were collected annually for 5 years (Time 1 to Time 5) using structured interviews and objective measurements of physical status. The current study was approved by the Research Ethics Board of the University of New Brunswick, Fredericton.

In this analysis, structural equation modeling (SEM) was used to test the proposed model. SEM is a statistical technique used to evaluate the integrity of a theory and has the advantage of permitting the differentiation of theoretical concepts (latent variables) from measured variables (indicators) [46]. Another advantage is the ability to examine direct and indirect pathways among interrelated concepts [47, 48]. We operationalized theoretical concepts using variables in the existing data set.

### Theoretical concepts and their operationalization

#### Cardiovascular disease risk

Because elevated blood pressure is often one of the first detectable signs of CVD especially in a non-clinical sample, we conceptualized it as an indicator of early CVD risk [49]. Two indicators of CVD risk were used in this model testing: systolic and diastolic blood pressure. Two systolic and diastolic blood pressure readings were obtained during Time 5 data collection. The averages of the two readings for systolic and diastolic pressure were used as separate indicators of CVD risk.

#### Lifetime abuse

Severity of lifetime abuse was defined as the degree of physical, sexual, and/or psychological abuse experienced by the women throughout their lifespan using data collected at Time 1. Although there were no indicators for severity of *lifetime* abuse within the data file, indicators of different types of abuse at different life stages were available (i.e., child abuse, partner abuse, sexual assault during adulthood).

##### Child abuse

Child abuse severity was operationalized using the abuse items of the Childhood Trauma Questionnaire (CTQ), a 28-item retrospective self-report measure of the incidence and frequency of abuse and neglect during childhood [50]. Women rated the frequency of emotional, physical and sexual abuse events on a 5-point rating scale from 1 (never true) to 5 (very often true), for a total summative child abuse score of 15 to 75, with higher scores indicating greater severity of abuse. In this study, the Cronbach’s alpha for the 15 abuse items was .94.

##### Partner abuse

The Index of Spouse Abuse (ISA) is a 30-item self-report measure of the severity of past physical, sexual, and psychological abuse from an intimate partner [51]. Using a 5-point rating scale with responses ranging from ‘never’ to ‘frequently’, women indicated the frequency that each abusive act was experienced. Predefined weights were applied to each item to reflect severity of abuse, and the product terms were summed for a total score (range 0–100), with higher scores indicating greater severity of abuse. Acceptable reliability and validity for the ISA have been demonstrated in samples of women [51, 52]. The Cronbach’s alpha in the current study was .91.

##### Adult sexual assault

The experience of adult sexual assault (ASA) was measured by one dichotomous question, “not including the relationship with the partner, have you ever been sexually assaulted as an adult? By sexual assault I mean did you ever have sexual contact against your will?” A score of 1 was assigned to an affirmative answer, and a score of 0 represented a negative answer.

#### CVD risk behaviors

Smoking history was operationalized by self-reports of current and past smoking behavior at Time 5; data were recoded to classify each participant as: “never smoked”, “past smoker”, and “current smoker”. These categories provide information about recency of nicotine exposure, with current smokers having the most recent exposure or the riskier behavior. The validity of self-reported smoking behavior to biochemical smoking levels has been demonstrated with a reported sensitivity of 92% and specificity of 98% [53, 54].

Overeating was conceptualized in the model as body weight with two indicators at Time 5: Body Mass Index (BMI) and Waist Circumference. BMI was computed by dividing a woman’s weight by the square of her height (kg/m2) [55]. Waist circumference is a measure of central obesity and provides a gauge of body fat distribution [56]. Greater BMI and waist circumference values represent greater CVD risk [55, 56].

#### Depressive symptoms

Depressive symptom severity was operationalized using the 20-item Center for Epidemiologic Studies of Depression scale (CES-D) [57]. Women rated the frequency of their past week symptoms on a 4-point rating scale, ranging from ‘rarely or none of the time’ [less than 1 day] to ‘most of the time’ [5–7 days]. To examine the change in depressive symptoms after leaving an abusive situation, summative CES-D scores for each of the 5 data collection periods were used (Time 1 to Time 5), with higher scores indicating greater symptomatology. The CES-D has demonstrated validity and reliability with Cronbach’s alphas ranging from .92 to .95 in samples of women with histories of abuse [58, 59]. The Cronbach’s alphas for the present study ranged from .93 to .95 across the five periods of measurement.

#### Age

Age is a well-established, non-modifiable risk factor for the development of CVD [60] and was defined as participants’ self-reported age in years at time of enrollment.

### Sample

A subset of 227 women with complete data was included in the analysis. Their mean age at Time 1 was 39.4 years (*SD* = 9.8; range 19–63). The majority self-identified as white, with only 13.2% identifying as a visible minority. They had completed an average of 13.5 years of education (*SD* = 2.5; range 7–22). Almost half (46.3%) were employed, whereas 30.8% received social assistance and 8.8% received disability support. Annual incomes ranged from $0 to $95,000 Canadian per year, with a mean income of $21,241 (*SD* = $18,259; median = $16,074). Over half of the women (56.4%) had a dependent child at home.

Women had been separated from the abusive partner on average 20.3 months (*SD* = 10.2; range 3–40). In terms of lifetime abuse, 85.5% reported experiencing emotional, physical, and/or sexual child abuse; 38.8% had experienced sexual assault from someone other than their ex-partner; and over half of the women (57.2%) had experienced abuse from more than one partner. Severity of abuse from the most recent partner was moderate to severe, with a mean ISA score of 53.0 (*SD* = 19.4; range = 18.2 to 100.0). The average duration of their relationship with the most current abusive partner was 8.9 years (*SD* = 8.1; range 0.3 to 37.0 years) and 38.8% reported that abuse was ongoing at the Time 1 despite having ‘left’ the partner. Table 1 presents the descriptive statistics for indicators in the model.

**Table 1:** Descriptive statistics for concepts and indicators in the model (*N* = 227)

Concepts & indicators	Mean (SD); Range
Age	
Age in Years	39.4 (9.8); 19.0–63.0
Child Abuse	
Childhood Trauma Questionnaire	35.6 (16.0); 15.0–75.0
Partner Abuse	
Index of Spouse Abuse Questionnaire	53.0 (19.4); 18.2–100.0
Adult Sexual Assault	
Adult Sexual Assault Question: %(n)	38.8% (*n* = 88)
Depressive Symptoms	
CES-D score Time 1	24.2 (13.0); 1.0–54.7
CES-D score Time 2	21.6 (13.5); 0.0–51.0
CES-D score Time 3	19.1 (13.1); 0.0–53.0
CES-D score Time 4	18.4 (13.0); 0.0–60.0
CES-D score Time 5	18.5 (13.0); 0.0–55.0
Smoking History	
Smoking Behavior: %(n)	
- Never smoked	33.0% (*n* = 75)
- Past smoker	30.0% (*n* = 68)
- Current smoker	37.0% (*n* = 84)
Body Weight	
Body Mass Index	27.8 (6.7); 15.0–50.4
BMI Categories^a^: %(n)	
Underweight	2.2% (*n* = 5)
Average weight	39.2% (*n* = 89)
Overweight	26.9% (*n* = 61)
Obese	31.7% (*n* = 72)
Waist Circumference	34.8 (6.5); 20.0–56.0
CVD Risk	
Systolic Blood Pressure	118.9 (15.5); 83.0–185.0
Diastolic Blood Pressure	74.3 (9.5); 49.0–113.0
Blood Pressure Categories^b^: %(n)	
Normal	50.2% (*n* = 114)
Prehypertension	39.2% (*n* = 89)
Hypertension	10.6% (*n* = 24)

*CES-D* Center for Epidemiologic Studies of Depression Scale

^a^Heart and Stroke Foundation BMI Categories

^b^JNC VII Blood Pressure Categories

Three statistically significant differences were observed between the characteristics of the 227 women included in this analysis and the total sample. One, significantly more women in the total sample identified as a visible minority; *X*^2^ (df 1, *n* = 303) =7.64, *p* < .01. Two, more women in the total sample reported experiencing physical abuse as a child; *X*^2^ (1, *n* = 300) =6.70, *p* = .01. Three, ISA scores were higher for the total sample (μ = 59.1, SD = 20.4) than for the study sample (μ = 53.0, SD = 19.4, *t (137.3)* = − 2.36, *p* = 0.02).

### Data analysis

Data were analyzed using SPSS and AMOS version 19®. Descriptive statistics were computed for all variables checking for outliers, violations in assumptions underlying the statistical tests, and overall accuracy of the data. Bivariate correlations were computed to explore the associations among study variables (Table 2). The sample of 227 was deemed adequate to test model fit according to guidelines recommended by Lee, Cai, and MacCallum [61]. For interpreting statistical significance of the pathways, the alpha was preset to .05.

**Table 2:** Pearson correlations among measured indicators in the model (*N* = 227)

		1	2	3	4	5	6	7	8	9	10	11	12	13
1.	AGE	1.00												
2.	CTQ	−.02	1.00											
3.	ASA	.04	.35^*^	1.00										
4.	ISA	−.19^*^	.34^*^	.16^*^	1.00									
5.	CESD-1	−.01	.32^*^	.13^*^	.28^*^	1.00								
6.	CESD-2	−.09	.27^*^	.14^*^	.18^*^	.60^*^	1.00							
7.	CESD-3	−.02	.37^*^	.27^*^	.21^*^	.48^*^	.58^*^	1.00						
8.	CESD-4	−.07	.34^*^	.16^*^	.31^*^	.46^*^	.56^*^	.64^*^	1.00					
9.	CESD-5	−.02	.17^*^	.13^*^	.17^*^	.42^*^	.49^*^	.54^*^	.54^*^	1.00				
10.	SMOKE	−.15^*^	.24^*^	.16^*^	.27^*^	.03	.07	.11	.13^*^	.09	1.00			
11.	WC	.09	.19^*^	.05	.07	.09	.06	.06	.01	.18^*^	−.06	1.00		
12.	BMI	.08	.15^*^	.04	.05	.07	.02	.02	−.03	.11	−.10	.89^*^	1.00	
13.	SBP	.40^*^	.05	.01	−.10	.06	−.08	−.11	−.09	−.05	−.05	.39^*^	.35^*^	1.00
14.	DBP	.24^*^	.04	−.02	−.10	.00	−.07	−.11	−.12	.02	−.05	.46^*^	.45^*^	.66^*^

*CTQ* Childhood Trauma Questionnaire (Emotional, Physical, Sexual), *ISA* Index of Spouse Abuse, *ASA* Adult Sexual Assault, *CESD* Center for Epidemiologic Studies Depression Scale measures 1 thru 5, *BMI* Body Mass Index, *WC* Waist Circumference, *SBP* Systolic Blood Pressure, *DBP* Diastolic Blood Pressure

^*^Significant at the *p* < .05 level

**Fig. 2:**
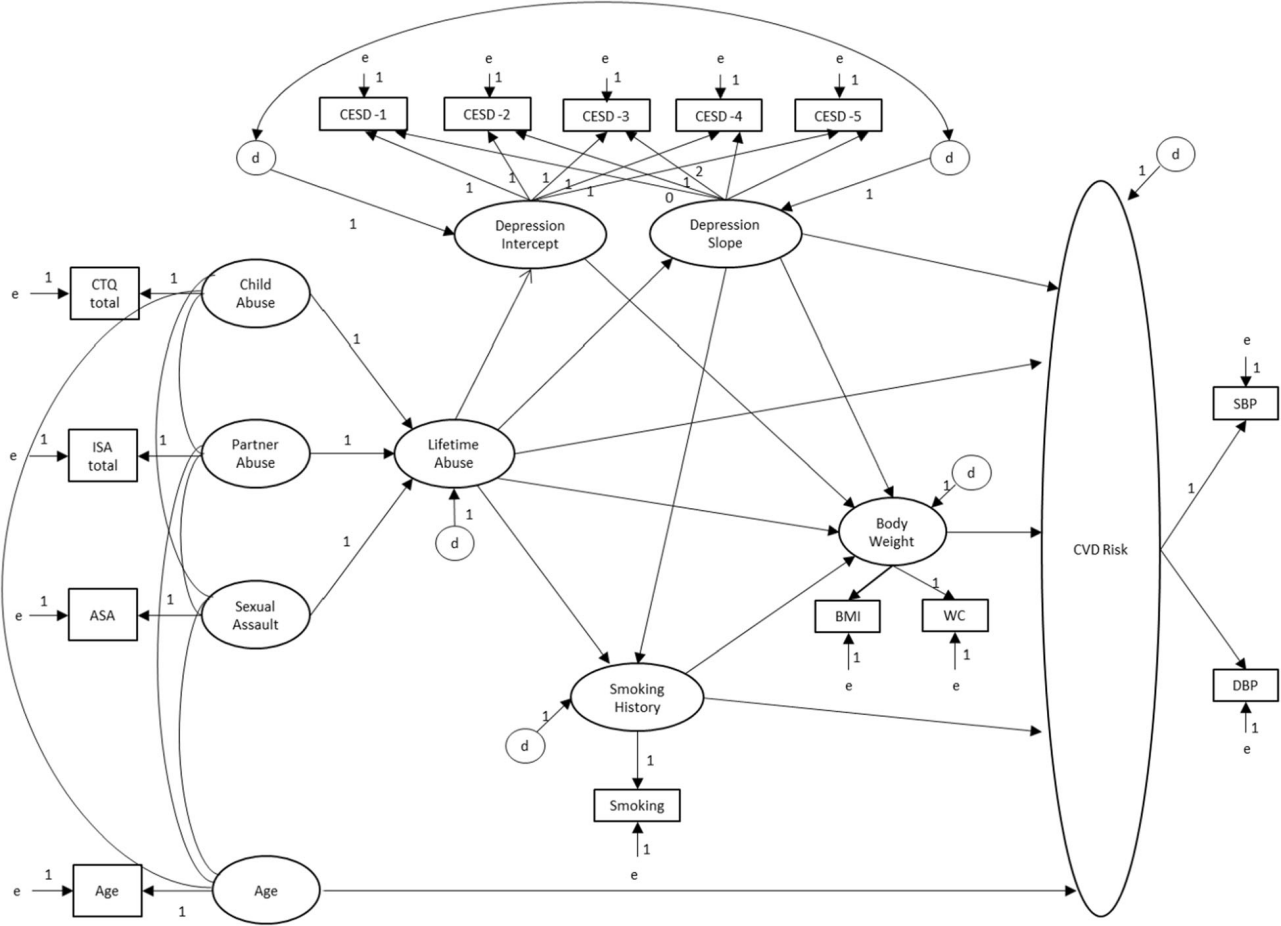
Hypothesized structural equation model with latent concepts and indicators. Larger circles/ovals represent the latent concepts; squares/rectangles represent the indicators; ‘e’ represent the amount of assigned measurement error; ‘d’ represent the disturbance; the arrows represent the direction of the hypothesized pathways; the numerical values next to the arrows indicate the scaling of the pathway; CTQ = Childhood Trauma Questionnaire; ISA = Index of Spouse Abuse; ASA = adult sexual assault; CESD = Center for the Epidemiologic Studies of Depression scale at data collection times 1 through 5; BMI = body mass index; WC = waist circumference; SBP = systolic blood pressure; DBP = diastolic blood pressure; CVD Risk = Cardiovascular disease risk

SEM, using maximum likelihood estimation, was conducted to test the direct effects of severity of lifetime abuse on CVD risk as well as its indirect effects through CVD risk behaviors (smoking and body weight) and through women’s initial level of depressive symptoms at Time 1 and the average rate of change in their depressive symptoms over time (Fig. 2).

To address the lack of an indicator of *lifetime* abuse within the data file, a phantom variable was created by drawing on the works of Rindskopf [62] and Hayduk [63, 64]. A phantom variable has no observed indicators rather it is defined by the concepts that have paths leading to it. Lifetime abuse was depicted in the model as a composite of childhood abuse, partner abuse, and adult sexual assault, each with its own indicator obtained from the Time 1 data collection period. Because the indicators for these concepts were measured on different scales, their scores were standardized so each indicator had a mean of 0 and standard deviation of 1. The phantom variable was scaled so that it was explained equally by the abuse concepts (i.e., each path assigned a value of 1). Specifying the model in this way allowed the variance of the phantom variable to be computed based on the variances (var) and covariances (covar) observed among the three abuse indicators (Variance _Phantom_ = Var_X1_ + Var_X2_ + Var_X3_ + 2Cov_X1,X2_ + 2Cov_X1,X3_ + 2Cov_X2,X3_).

The amount of error assigned to the self-reported measures (ISA, CTQ, and CES-D) was based on the observed reliability (1 – Cronbach’s alpha). For Adult Sexual Assault, given the dichotomous nature of the question, 10% measurement error was assigned. Twenty percent error was assigned to the phantom variable to reflect the absence of a direct indicator of this concept and the possibility of other sources of abuse. Smoking was assigned 5% error reflecting the reported sensitivity and specificity of self-reported behaviors [54]. Waist circumference, body mass index, and systolic and diastolic blood pressure were all assigned 10% error reflecting challenges inherent in obtaining measurements within the home setting (e.g., noise interference with blood pressure auscultation, clothing interference with waist circumference measurement). Finally, 1% error was assigned to age because date of birth was collected at each of the five data collections, allowing for quality control checks.

To examine the effects of women’s Time 1 depressive symptoms on CVD risk and the observed rate of change in women’s depressive symptoms over the 5 years on their CVD risk, an application of SEM called Latent Growth Curve Modeling was used [65]. The intercept reflected women’s initial level of depressive symptoms (at Time 1) and the slope represented the rate of change in depressive symptoms over the five data collection periods. The paths from the intercept to each of the five measures of depressive symptoms were fixed at a constant of 1, representing the initial level of depressive symptoms. For the slope, the descriptive statistics for the measures of depression suggested the rate of change may not be consistent across all time periods, therefore we chose to free some of the parameters. As such the path coefficients from Depression Slope to Time 1, 2 and 3 measures were fixed at 0, 1, and 2 respectively to reflect a constant rate of change over time, whereas the path coefficients from the Slope to the Time 4 and Time 5 indicators were left free to be estimated. It is not possible to model correlations between endogenous variables (variables whose values are determined by other variables) within the SEM model. To acquire this information a correlation pathway was drawn between the disturbances for the Depression, Slope and Intercept [66, 67]. Disturbances represent all other influences on the latent variable other than those shown in the model (i.e., unexplained variance) [47]. It is reasonable to assume that these influences would be similar for the two concepts.

Model fit was evaluated using the following indices: chi-square (*X*^2^) (desired *p*-value >.05), the Root-Mean Square Error of Approximation (RMSEA; < .05 represents a good fit while <.08 represents an acceptable fit), the Comparative Fit Index (CFI; desired value >.90) and the Tucker-Lewis Index (TLI > .90) [47, 68]. To provide evidence of the stability of the estimated model parameters, bootstrapping was conducted for the final model and the 95% confidence intervals assessed.

## Results

**Fig. 3:**
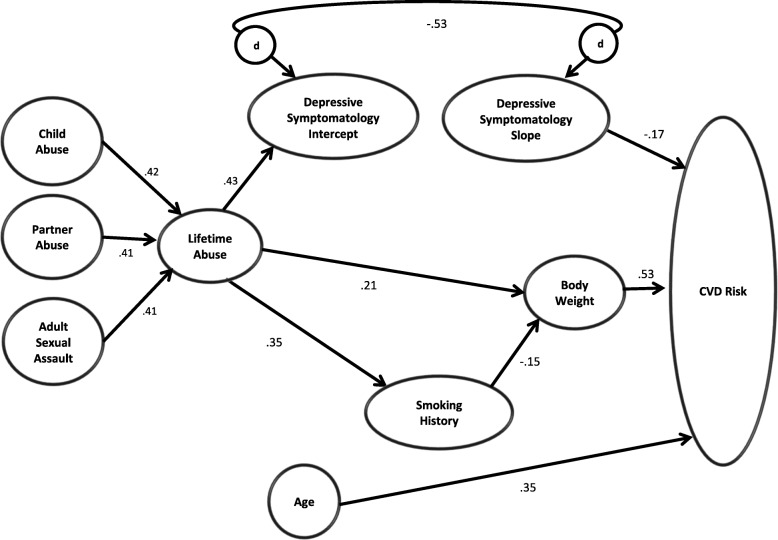
Statistically significant standardized parameter coefficients for the tested model. Solid lines denote statistically significant pathways at *p* < .05. Paths from Child Abuse, Partner Abuse, and Adult Sexual Assault are all fixed

Results of the initial model testing revealed a poor fit between the model and the data. Standardized residuals and modification indices were examined to identify problematic areas of the model and possible areas for improvement. The model was examined and an incremental process used to make changes one at a time, with the model run after each change allowing for the reassessment of fit. A theoretically justifiable structural change was made to the model; a pathway was added from Smoking History to Body Weight. Adjustments were also made to the amount of error assigned. The amounts of error for the indicators were increased as follows: 15% for both ASA and ISA, 30% for CES-D, 16% for BMI, and 25% for both systolic and diastolic blood pressures, with a 5% decrease for waist circumference. After the aforementioned adjustments, the fit indices indicated the model adequately fit the data (Table 3). The Relative Chi Square with 1 degree of freedom was 1.99; a value less than 2.0 suggesting the model fits the data reasonably well [69]. Figure 3 depicts the statistically significant paths within the model, whereas Table 3 presents the standardized parameter coefficients and the 95% confidence intervals for the standardized regression weights generated by the Bootstrapping.

**Table 3:** Standardized coefficients for the tested model (*N* = 227)

Outcomes and predictors	Standardized causal effects			Bootstrapped 95% confidence intervals for standardized regression weights
	Direct	Indirect	Total	
Depressive Symptoms Intercept (*R*^2^ = .18)				
Lifetime Abuse	.428^*^	–	.428	[.254, .585]
Depressive Symptoms Slope (*R*^2^ = .000)				
Lifetime Abuse	−.020	–	−.020	[−.247, .213]
Smoking History (*R*^2^ = .14)				
Lifetime Abuse	.349^*^	−.002	.346	[.204, .487]
Depressive Symptoms Slope	.122	–	.122	[−.100, .316]
Body Weight (*R*^2^ = .05)				
Lifetime Abuse	.213^*^	−.048	.165	[.023, .402]
Depressive Symptoms Intercept	.012	–	.012	[−.180, .223]
Depressive Symptoms Slope	.031	−.019	.012	[−.210, .295]
Smoking History	−.153^*^	–	−.153	[−.296, .005]
CVD Risk (*R*^2^ = .41)				
Lifetime Abuse	−.135	.121	−.013	[−.314, .056]
Depressive Symptoms Slope	−.167^*^	.017	−.149	[−.350, .040]
Smoking History	.091	−.080	.011	[−.053, .226]
Body Weight	.526^*^	–	.526	[.392, .620]
Age	.347^*^	–	.347	[.212, .453]
Model Fit Indices				
Indices	Desired	Actual		
χ^2^	*p* > .05	148.94 (*df* = 75) *p* < .001 or 1.99 (df = 1)		
Comparative Fit Index (CFI)	>.90	.94		
Tucker-Lewis Index (TLI)	>.90	.92		
Root Means Square of Approximation	<.08 acceptable	.07		
Standardizes Residuals	None > 2.0	Largest + 1.90		

Due to rounding, total effects may not equal sum of direct and indirect effects

^*^p < .05. Dashes indicate no indirect pathway was specified

Although it was hypothesized that severity of Lifetime Abuse would have a significant effect on both the severity of depressive symptoms at Time 1 and the average rate of change in depressive symptoms over time, only one of the hypothesized pathways was supported. Eighteen percent of the variance in the intercept for Depressive Symptoms was explained by its sole predictor of Lifetime Abuse (β = .43). Consistent with our prediction, the positive beta suggests that women with more severe lifetime abuse histories tended to have higher levels of depressive symptoms at Time 1. Despite its significant effect on Time 1 level of depression, Severity of Lifetime Abuse did not have a significant effect on the observed change in depressive symptoms over time. The average change (i.e., Slope) in depressive symptoms between each time of measurement was −.25 and statistically significant (*p* < .001), which suggests that on average women’s depressive symptoms improved with time. However, the estimated standardized parameter coefficients from the Slope to the five CES-D measures support a non-linear rate of change (.00, .31, .63, .68, .70 [the first three paths were fixed]). A negative correlation (β = −.53) between the disturbances (unexplained variances) for the Intercept and the Slope suggests that women who had higher initial levels of depressive symptoms tended to experience less change in symptoms during the study period.

Severity of Lifetime Abuse positively affected CVD risk behaviors, but contrary to prediction severity of depressive symptoms did not. Approximately 14% of the variance in Smoking History was explained by the model. The pathway from Lifetime Abuse was statistically significant (β = .35), suggesting that women with more severe abuse histories tended to have more recent exposure to nicotine. The model explained 5% of the variance in Body Weight with two statistically significant paths. The strongest causal path was a positive one from Lifetime Abuse (β = .21), indicating that women with more severe abuse histories were more likely to be overweight at Time 5. The second path from Smoking History to Body Weight was negative (β = −.15), suggesting that women who weighed less tended to have more recent exposure to nicotine. Neither the Time 1 level of depressive symptoms nor the change in depressive symptoms over time had a statistically significant effect on Smoking Behavior or Body Weight.

Finally, approximately 41% of the variance in CVD risk was explained by the model with three statistically significant paths. The strongest path was from Body Weight (β = .53), suggesting that women with greater body weight tended to have greater CVD risk. A second positive path was from Age (β = .35), indicating that older women tended to have greater CVD risk. The third path, was from Depression Slope, with a negative coefficient (β = −.17) signifying that women who experienced less change in their depressive symptoms over time tended to have greater CVD risk. Contrary to hypothesis, the direct path between Lifetime Abuse and CVD risk was not statistically significant (β = −.13; *p* = .08).

### Model stability

Bootstrapping was successfully completed with 500 samples. The computed 95% confidence intervals suggest potential instability in the parameter estimates for 2 of the 13 model paths: (1) going from smoking history to body weight (95% CI −.296 to .005) and (2) going from depression slope to CV risk (95% CI −.350 to .040).

## Discussion

Descriptively, in terms of the CVD risk behaviors and risk factors exhibited by the sample, these women were at above average ‘risk’ (see Table 1). Thirty-seven percent of women were current smokers at Time 5, which is a little over double the national average for Canadian women [70]. Approximately 59% of the women at Time 5 were categorized as being overweight or obese according to their BMIs, which is also higher than the average for Canadian women of approximately 53% [71]. Further, whereas only 10% of women met the criteria for hypertension at Time 5, 39% fell within the ‘pre-hypertension’ range according to the Joint National Committee on Prevention, Detection, Evaluation and Treatment of High Blood Pressure guidelines, a rate that is almost two and a half times what is observed in the general population for women [72]. Empirical research suggests that individuals who have blood pressures within the pre-hypertensive range are at twice the risk of developing hypertension as those with lower values [49], therefore identification of women who are at risk for developing hypertension is clinically important to permit early intervention. In terms of depressive symptoms, at Time 1 almost 69% of women had clinically significant levels of depressive symptoms determined by the CES-D cut off value of 16, whereas the percentage decreased to 53% at Time 5. While the decrease in depressive symptoms over time is encouraging, this percentage is still notably high considering that women had been out of the abusive relationship for 5 to 8 years and given that the rate of major depression within the previous 12 months for women in the general Canadian population is approximately 5.9%, with a lifetime prevalence rate of approximately 15.1% [73]. Collectively these findings signify a worrisome CVD risk trend among women with histories of abuse.

Results of the tested model provide preliminary evidence supporting many of the hypothesized pathways by which severity of lifetime abuse can increase CVD risk (as indicated by measures of systolic and diastolic blood pressure) among women. Findings provide support for the notion that CVD risk behaviors of smoking and overeating increase with severity of lifetime abuse and that these behaviors are present 5 to 8 years after leaving an abusive relationship. This extends the previous work of Scott-Storey et al. [24] in which severity of intimate partner violence was positively associated with smoking behavior and greater body mass index in the immediate aftermath of separating from an abusive partner. Addition of the model-specified pathway between smoking history and body weight suggests current smokers may weigh less than those who either never smoked or who were past smokers. This is consistent with evidence supporting that smokers tend to weigh less than non-smokers, likely as a result of the increased energy expenditure and the appetite suppressing effect of nicotine [74]. However, the result is a paradox; the total effect of severity of lifetime abuse on CVD risk is partially reduced (mediated) by women’s smoking history. In other words, the negative path weakened the total effect of severity of lifetime violence on CVD risk. Although possibly a spurious finding, it may also be a result of the relatively young mean age of the sample (39.4 years) in terms of the negative effects of nicotine exposure on blood pressure. Replication of the model with a more age-diverse cohort of women would better illuminate the relationship between smoking history, lifetime abuse and CVD risk. Additionally, in the current study smoking was measured as ‘current, former, or never’; future studies would benefit from including other indicators of smoking intensity such as duration, frequency and amount to better predict CVD risk.

Contrary to hypothesis, neither the Time 1 level of depressive symptoms, nor the change in depressive symptoms over time, had an effect on smoking behavior or body weight. This finding suggests that among women with abuse histories, it is the severity of lifetime abuse that determines the adoption and persistence of these particular CVD risk behaviors, not the presence of or change in depressive symptoms. The lack of a significant pathway is surprising given the body of research that supports the relationships between depression and smoking and between depression and body weight [10, 23, 37, 43]. Regardless, this finding should not be seen as evidence to suggest clinicians stop paying attention to depressive symptoms in the context of behavior *modification* as research has shown that depressive symptoms influence the degree to which a person can change maladaptive behaviors and adhere to medical treatment regimens [31]. Therefore, success in reducing CVD risk behaviors may be mitigated unless depressive symptoms are addressed.

A finding that warrants consideration is that the severity of depressive symptoms indirectly influenced the effect of the severity of lifetime abuse on CVD risk. Women with more severe lifetime abuse histories tended to have higher levels of depressive symptoms in the initial period after leaving the abusive relationship (intercept), highlighting severity of abuse as a factor influencing the severity of depressive symptoms. Further, it suggests that women who experience the greatest cumulative lifetime abuse are at greatest risk for depression post-separation and that these women may potentially benefit from early intervention. However, a common assumption that leaving equates to an end of stress and a resolution of health issues leads to few health resources being allocated to women who separate; our findings challenge this belief by providing further evidence of sustained poor mental health in the early years after leaving an abusive relationship.

Notably, severity of lifetime abuse did not directly affect change in depressive symptoms over time (slope). This could be interpreted as an indication that despite cumulative lifetime abuse severity affecting initial depressive symptom severity after leaving an abusive relationship, it does not affect the potential for change in depressive symptoms over time. This is an encouraging finding as it contradicts perceptions that individuals with severe cumulative abuse histories may be permanently ‘damaged’ and suggests that physiological changes resulting from abuse that affect depression can be mitigated regardless of the severity of the abuse history. In other words, this research provides beginning evidence that change is possible and depressive symptoms can improve for women with even the most severe lifetime abuse histories, signifying the importance of clinicians helping women to manage their depressive symptoms in the aftermath of leaving an abusive partner.

Women with the most severe depressive symptoms in the early years after leaving tended to experience the *least* change in symptoms over the study period (negative correlation between disturbances; −.53). This finding is a new contribution to the literature. It highlights that women suffering from high levels of depressive symptoms in the immediate aftermath after leaving may be most at risk for persistent mental illness post-separation and reinforces the need for early depression intervention/treatment after leaving the abusive relationship. It is also possible that women who experience *less change* in their depressive symptoms over time may have higher CVD risk, highlighting this group of women as an potential target group for primary and secondary prevention of CVD.

The longitudinal nature of the current study addresses current gaps in knowledge related to depressive symptoms after leaving an abusive relationship. Findings suggest that although most women experience a reduction in depressive symptoms after leaving an abusive relationship, the rate of change/improvement appears to subside over time (i.e., nonlinear). Initial improvement in depressive symptoms may lead clinicians to falsely assume that depression resolves with time; however, our findings suggest that despite initial improvement, a significant proportion of women continue to experience significant levels of depressive symptoms years after leaving, warranting clinical attention. Further, improvements in depressive symptoms appeared to level off after a few years, which raises questions as to whether this leveling can be prevented with appropriate mental health interventions targeted in the period after leaving an abusive relationship.

Contrary to prediction, severity of lifetime abuse did not have a statistically significant direct effect on CVD risk as indicated by measures of systolic and diastolic blood pressure. However, it may be premature to dismiss this pathway. It is possible that this direct path encompasses too much in terms of a complex array of stress related neuroendocrine and physiological responses within the body (e.g., cortisol, epinephrine, inflammatory changes) through which abuse affects CVD risk and may include other influencing factors that do not operate through depression or risk behaviors); therefore the ability to detect a significant path might be enhanced by teasing out some of these influences. For example, future research might benefit from including biophysical (e.g., cortisol, C-reactive protein) and psychosocial (e.g., posttraumatic stress disorder) measures over time. Alternatively, the study may not have been sufficiently powered to attain statistical significance for some of the weaker but noteworthy paths.

Overall, our findings reveal that there are multiple pathways through which abuse can affect CVD risk, suggesting the possible utility of a multimodal approach to intervention focusing concurrently on chronic stress (abuse history), depressive symptoms, and CVD risk behaviors to best optimize cardiovascular health and reduce CVD risk among women who have experienced abuse. Importantly, the model was supported by the data, explaining 41% of the variance in CVD risk among a group of women who had left an abusive partner.

In understanding the implications of abuse on women’s health, a limitation of previous research has been in examining singular types of abuse or only recent experiences of abuse; our findings suggest that severity of *lifetime* abuse increases women’s CVD risk indirectly through various pathways. Implications of these findings are particularly salient as they contribute to our evolving theoretical understanding of cumulative lifetime abuse, and support the need for both clinicians and researchers to consider severity of lifetime abuse as a contributor to CVD. This assertion is supported by the lifetime abuse profile of women within this study, which although staggering, was not unexpected.

### Strengths and limitations

Despite notable methodological challenges inherent in the study of lifetime abuse, including the lack of instruments that measure cumulative abuse across the lifespan, we were able to offer one potential approach for addressing this challenge by operationalizing lifetime abuse severity as a phantom variable within the SEM. The use of a phantom variable allowed us to create a concept that was a composite of child abuse, partner abuse, and adult sexual assault; its successful inclusion in the model provides justification for further investigation with other types of abuse encountered throughout the lifespan (e.g., dating violence, harassment, stalking, work place bullying). As the understanding of the phenomenon of cumulative lifetime abuse evolves, researchers need to consider alternative ways of conceptualizing and operationalizing its complexity, including the development of measurement instruments.

To our knowledge, this is the first study to empirically test *cumulative lifetime* abuse as a contributing factor for CVD, with the model itself being grounded in the premise that multiple factors interact to influence CVD risk. The longitudinal design provides insight into some of the pathways through which abuse can affect CVD risk over time. Further, SEM is one of the few statistical procedures that acknowledges imperfections in measurement tools by allowing researchers to give an indication of the extent of measurement error. Although cut-points used to evaluate model fit in this analysis are consistent with current norms, the criteria for interpreting model fit are currently the focus of debate [75].

Study findings should be interpreted alongside several limitations. Abuse experiences were measured using retrospective self-reports, which often come with criticisms of inaccurate and biased recall; however, it is generally accepted that people are more likely to underestimate actual occurrences of abuse rather than overestimate [76, 77]. Additionally, the analysis was conducted using an existing data set, which inherently poses challenges. For example, the data were originally collected to broadly examine women’s mental and physical health in the early years after leaving an abusive relationship and therefore, were not collected specifically to examine CVD risk. As a result, important indicators such as biophysical measures of chronic stress (e.g. cortisol, inflammatory markers) and objective measures of CVD (e.g., atherosclerosis) were not included; their inclusion in future research may improve model fit. Future research would also benefit from using repeated measures of blood pressure over time to capture a more reliable indicator of CVD risk, rather than a one-time value. Further testing with a more age diverse cohort of women might enhance the ability to explain CVD risk and enhance the predictive ability of the model.

It is acknowledged that important factors/determinants known to influence mental and cardiovascular health were not included in the tested model (e.g., socioeconomic status, resiliency, personal resources, social support, professional support, cardiovascular medications, current medical history, menopausal status, CVD family history, alcohol and substance use, and other CVD risk behaviors, such as physical inactivity) [78–80]. Inclusion of these factors might improve predictive ability of the model, the variance explained, and model fit. Because Posttraumatic Stress Disorder (PTSD) is often co-morbid with depression among women with histories of abuse and is receiving attention as an independent risk factor for CVD [10, 81, 82], future research examining PTSD’s unique and additive effects with depression would add to our understanding of the role of mental health in CVD risk. Additionally, while the sample size was determined to be adequate for initial model testing, future model testing using a larger sample is needed to validate the model and the parameter estimates as well as test alternative or competing models [47].

## Conclusion

Results of this model testing provide preliminary evidence to support Scott-Storey’s [18] conceptual model in terms of the physiological, psychological, and behavioral paths through which severity of lifetime abuse can affect CVD risk among women who have left an abusive relationship. The model fit the data reasonably well, offering new insight into the interplay among lifetime abuse, CVD risk behaviors, level of depressive symptoms at different points in time, and CVD risk. Findings have implications for clinicians working with women in the period after leaving an abusive relationship including the potential utility of multimodal interventions focused concurrently on abuse history, mental health, and risk behaviors in reducing CVD risk and optimizing cardiovascular health. Our findings also contribute to the growing recognition of the long-term effects of lifetime abuse on cardiovascular health and provide a novel approach for studying the concept of cumulative lifetime abuse through the use of a phantom variable.

## Data Availability

The dataset used in the analyses reported here are not publicly available. Access to these data was provided by Dr. Marilyn Ford-Gilboe (PI of the primary study) under a limited agreement. Requests to access these de-identified data can made to Dr. Marilyn Ford-Gilboe (mfordg@uwo.ca) who will consider the request and may grant reasonable access.

## Abbreviations

ASA: Adult sexual assault
BMI: Body mass index
CES-D: Center for epidemiologic studies of depression scale
CFI: Comparative fit index
CTQ: Child trauma questionnaire
CVD: Cardiovascular disease
ISA: Index of spouse abuse
JNC VII: Joint national committee on prevention, detection, evaluation and treatment of high blood pressure guidelines
RMSEA: Root-mean square error of approximation
SEM: Structural equation modeling
TLI: Tucker-Lewis index
